# Prognostic biomarkers of Parkinson’s disease in the Spanish EPIC cohort: a multiplatform metabolomics approach

**DOI:** 10.1038/s41531-021-00216-4

**Published:** 2021-08-16

**Authors:** Carolina Gonzalez-Riano, Jorge Saiz, Coral Barbas, Alberto Bergareche, José Mª Huerta, Eva Ardanaz, Marcela Konjevod, Elisabet Mondragon, M. E. Erro, M. Dolores Chirlaque, Eunate Abilleira, Fernando Goñi-Irigoyen, Pilar Amiano

**Affiliations:** 1 Centro de Metabolómica y Bioanálisis (CEMBIO), Facultad de Farmacia, Universidad San Pablo-CEU, CEU Universities, Urbanización Montepríncipe, Boadilla del Monte, Madrid, Spain; 2 Neurodegenerative Disorders Area, Biodonostia Health Research Institute, San Sebastián, Spain; 3 Disorders Unit, Department of Neurology, University Hospital Donostia, San Sebastián, Spain; 4 Biomedical Research Networking Centre Consortium for the Area of Neurodegenerative Diseases (CIBERNED), Madrid, Spain; 5 Instituto Murciano de Investigación Biosanitaria (IMIB), Murcia, Spain; 6 CIBER de Epidemiología y Salud Pública (CIBERESP), Madrid, Spain; 7 Instituto de Salud Pública de Navarra, Pamplona, Spain; 8 Division of Molecular Medicine, Rudjer Boskovic Institute, Zagreb, Croatia; 9 Department of Neurology, Complejo Hospitalario de Navarra, IdiSNA (Navarra Institute for Health Research), Pamplona, Spain; 10 Public Health Laboratory in Gipuzkoa, Biodonostia Health Research Institute, San Sebastián, Spain

**Keywords:** Metabolomics, Prognostic markers, Metabolomics, Neuroscience

## Abstract

The lack of knowledge about the onset and progression of Parkinson’s disease (PD) hampers its early diagnosis and treatment. Metabolomics might shed light on the PD imprint seeking a broader view of the biochemical remodeling induced by this disease in an early and pre-symptomatic stage and unveiling potential biomarkers. To achieve this goal, we took advantage of the great potential of the European Prospective Study on Nutrition and Cancer (EPIC) cohort to apply metabolomics searching for early diagnostic PD markers. This cohort consisted of healthy volunteers that were followed for around 15 years until June 2011 to ascertain incident PD. For this untargeted metabolomics-based study, baseline preclinical plasma samples of 39 randomly selected individuals that developed PD (Pre-PD group) and the corresponding control group were analyzed using a multiplatform approach. Data were statistically analyzed and exposed alterations in 33 metabolites levels, including significantly lower levels of free fatty acids (FFAs) in the preclinical samples from PD subjects. These results were then validated by adopting a targeted HPLC-QqQ-MS approach. After integrating all the metabolites affected, our finding revealed alterations in FFAs metabolism, mitochondrial dysfunction, oxidative stress, and gut–brain axis dysregulation long before the development of PD hallmarks. Although the biological purpose of these events is still unknown, the remodeled metabolic pathways highlighted in this work might be considered worthy prognostic biomarkers of early prodromal PD. The findings revealed by this work are of inestimable value since this is the first study conducted with samples collected many years before the disease development.

## Introduction

Parkinson’s disease (PD) is a neurodegenerative disorder characterized by a loss of dopaminergic neurons in the substantia nigra and the production of Lewy bodies. Such neurological alterations cause the motor and cognitive impairments^1–3^. Motor symptoms include bradykinesia, resting tremor, rigidity of muscles, problems with balance, and postural deformities^2–4^. Due to neurodegeneration, non-motor symptoms are also observed in individuals with Parkinson’s disease, which is characterized by behavioral changes, sensory abnormalities, autonomic dysfunction, sleep disturbances, dementia, psychosis, depression, anxiety, anhedonia, pain, and fatigue^2,3,5,6^. PD is a chronic and progressive disorder, with immunological, genetic, and environmental etiology^2^. Together with Alzheimer’s disease, it is one of the most prevalent neurodegenerative disorders with around 2% of affected people older than 60 years^2,7^. Diagnosis of PD is established according to the presence of parkinsonian motor symptoms, while therapy is exclusively symptomatic and cannot stop or slow down progression^4^. Since typical PD motor symptoms appear when there is already more than 80% of dopaminergic loss, identification of biomarkers for early diagnosis might drastically change diagnosis and treatment approaches^8^. Several approaches have already been proposed for the early diagnosis of PD, including positron emission tomography (PET) and single-photon emission computed tomography (SPECT) imaging, examining olfactory alterations that usually appear before any known motor symptoms, skin and colonic biopsy, gene sequencing, and changed metabolites, including uric acid, glutathione, and α-synuclein^2^. However, these approaches fall short for early diagnosis due to the complexity of PD. Therefore, currently novel diagnostic approaches that would combine several methods are necessary^2^. Moreover, a pre-symptomatic population has never been explored searching for biomarkers.

In this regard, metabolomics can be considered as a well-defined approach to unveil potential metabolic biomarkers to diagnose the disease when no PD symptoms have yet developed, to understand better its early pathophysiological mechanisms, to identify potential novel drugs, and to monitor the therapeutic outcome^9^. Numerous original metabolomics studies have been conducted using different biological samples in order to discover potential biomarkers and altered metabolic pathways in PD. This suggests that various metabolic pathways are associated with PD development and pathogenesis. Using different analytical techniques, alterations in these metabolic pathways were observed in the early, mid, and advanced stages of Parkinson’s disease. Altered amino acids, glycine, tryptophan, phenylalanine, leucine, and isoleucine metabolism, dysregulation of the TCA cycle, altered fatty acid, purine, and dopamine metabolism are involved in PD pathogenesis^10–13^. Metabolites involved in glycine and phenylalanine metabolism have an important role because of their association with dopamine. This metabolite is involved in the pathogenesis, especially in the early stage of PD^11,12^, while alterations in tryptophan metabolism have been associated with mitochondrial dysfunction, psychiatric symptoms, and altered brain metabolism^11,14^. The involvement of mitochondrial dysfunction and oxidative stress might have an effect on fatty acid metabolism, causing perturbations that are reflected in changed levels of fatty acids^10,11,15^. The implication of oxidative stress in PD development^16^ has also been reflected by altered levels of certain metabolites i.e antioxidants or markers of oxidative stress, including glutathione disulfide, ergothioneine, 8-hydroxyguanosine, 8-hydroxy-2′-deoxyguanosine, and bilirubin/biliverdin ratio^13,17–19^. Recently, an LC–MS metabolomics-based study conducted with plasma samples collected from PD patients reflected alterations in many metabolite classes, including a remarkable reduction of the levels of several free fatty acids, *cis*-aconitic acid, and an increment on the levels of several bile acids in the PD cohort, among others changes^20^. Together with other altered metabolic pathways, energy metabolism through the TCA cycle is associated with PD development. It is assumed that such alteration might be a result of a complex gene × environment interaction^13^. Due to the complexity of PD, it is assumed that several interconnected metabolic pathways are involved in symptom development and disease progression.

**Fig. 1:**
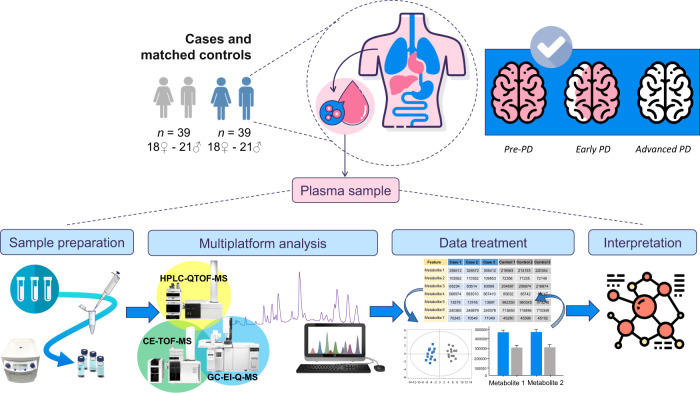
Metabolomics workflow followed for this study. Experimental design and the multiplatform untargeted metabolomics workflow followed for this study, including the sample preparation, multiplatform analysis (LC–MS, GC–MS, CE–MS), data treatment, and data interpretation.

Here, we used mass spectrometry (MS) coupled to gas chromatography (GC), liquid chromatography (LC), and capillary electrophoresis (CE) to obtain a metabolome coverage as extensive as possible, aiming the analysis of the global metabolic changes in the plasma samples from participants from the European Prospective Study on Nutrition and Cancer (EPIC) that were followed for almost 15 years. None of the participants had developed PD or showed any related symptoms at the time of sample collection. During the time they were followed up, some of them developed PD (pre-PD) while others did not (controls) (Fig. 1). Consequently, these samples are of extraordinary value for discovering potential biomarkers for the early diagnosis of PD, conferring this metabolomics-based study a unique advantage in the field.

## Results

### Metabolite coverage by a multiplatform untargeted metabolomics-based approach

**Fig. 2:**
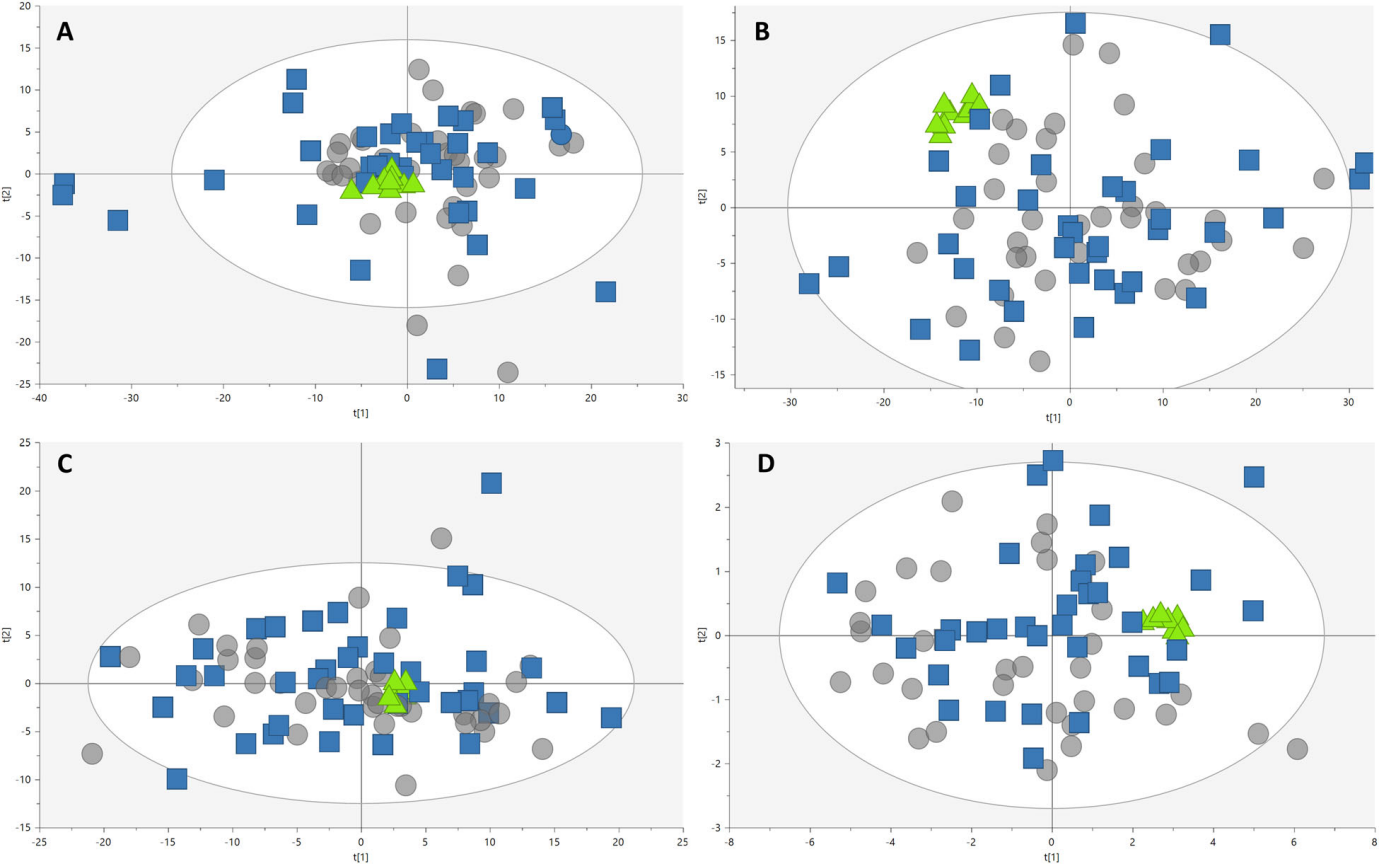
PCA-X score plot for the three analytical platforms. **A***R*^2^ = 0.396 represent the PCA‐X model for LC–MS ESI (+) (UV scaling); **B** *R*^2^ = 0.509 represent the PCA‐X model for LC–MS ESI (−) (UV scaling); **C** *R*^2^ = 0.356 represent the PCA‐X model for CE–MS (UV scaling); and **D** *R*^2^ = 0.691 represent the PCA‐X model for GC–MS (Par-log scaling). The four models showed very good QC clustering, thereby indicating good system stability and reliability of the results (gray circles, pre-PD; blue squares, controls; green triangles, QCs).

**Fig. 3:**
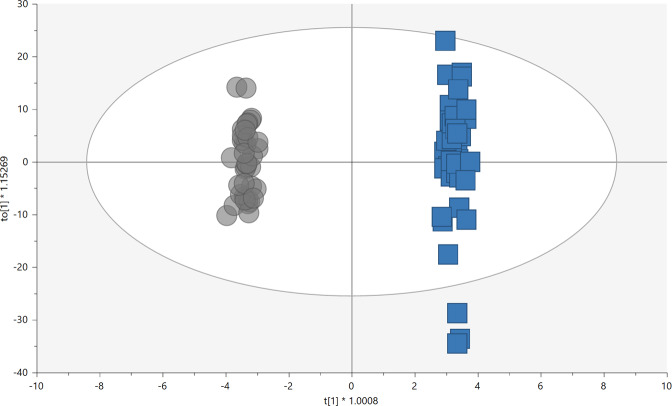
Supervised OPLS‐DA plot of LC–MS (+) data analysis. The model presented good quality of variance explained and predicted variance (*R*^2^ = 0.996, *Q*^2^ = 0.687) (UV scaling), and a *p* CV-ANOVA of 1.31 × 10^−8^ (gray circles, pre-PD; blue squares, controls).

The metabolic fingerprint of plasma samples collected several years before the development of symptoms of PD was achieved by performing a multiplatform untargeted metabolomics strategy. The metabolic alterations unveiled in this study indicate that the pathological mechanisms behind the onset and progression of PD starts many years before the appearance of the first PD symptoms. A significant amount of information was obtained from the multiplatform analysis, resulting in 672 and 571 metabolic features detected in LC–MS operated in positive and negative ionization modes, respectively, 332 signals were acquired from CE–MS and 104 from GC–MS analysis. After raw data matrix normalization, curation, and statistical analysis, 15, 12, 9, and 3 metabolites were detected as significantly affected by LC–MS ESI (−), LC–MS ESI (+), GC–MS, and CE–MS, respectively (Table 1). The cis-aconitic acid detected by GC–MS, and 2′-deoxyuridine 5′-monophosphate (dUMP) and serotonin both detected by CE–MS presented a *p*-value higher than 0.05. However, they displayed important changes when compared to the pre-PD group with healthy controls. Therefore, we will discuss about them later to enrich the biological interpretation of the results. After data normalization, all the PCA plots obtained displayed a tight clustering of the QC samples, revealing that the instrumental variation detected was corrected (Fig. 2). Although the plasma samples were collected when none of the subjects presented any PD symptoms, a supervised OPLS-DA model was obtained for the LC−MS ESI(+) data with great quality parameters values of explained variance (*R*^2^ = 0.996) and predicted variance (*Q*^2^ = 0.687)^21^. Finally, validation of the model was performed with cross-validation and CV-ANOVA tool provided by SIMPA-P+ software (*p* CV-ANOVA = 1.31 × 10^−^^8^) (Fig. 3). The metabolites presenting variable importance in projection (VIP) ≥1 and jackknifing confidence interval, not including the zero value were selected as statistically significant from the OPLS‐DA model.

**Table 1:** Metabolites that showed statistical significance when comparing PD subjects and healthy controls.

Metabolite	Mass	RT (min)	RMT	Technique	Error (ppm)	Adduct	CV (% in QC)	% change	*p*-value	VIP
Benzene and substituted derivatives										
2-Aminobenzoic acid	137.0476	15.47	0.90	CE–MS	8	[M+H]^+^	2.7	−40.1	0.012	–
Carboxylic acids and derivatives										
*Cis*-aconitic acid	174.0164	15.72	–	GC–MS	–	–	12.5	−23.1	*0.056*	–
3-Methoxytyrosine	211.0844	29.85	1.73	CE–MS	1	[M+H]^+^	4.3	16.0	0.026	–
Pipecolic acid	129.0789	15.15	0.88	CE–MS	6	[M+H]^+^	4.3	−52.1	0.024	–
Fatty acyls										
2-Hydroxy-3-methylbutyric acid	118.0629	8.30	–	GC–MS	–	–	4.5	−29.5	0.037	–
CMPF	240.0998	7.08	–	LC–MS ESI (±)	1	[M−H]^−^	3.6	31.7	0.035	–
Arachidonic acid	304.2402	31.02	–	LC–MS ESI (−)	8	[M+FA−H]^−^	4.1	−16.1	0.049	–
Docosapentaenoic acid (DPA)	330.2555	28.29	–	LC–MS ESI (−)	1	[M–H]^−^	4.2	−20.3	0.041	–
Linoleic acid	280.2402	20.38	–	GC–MS/LC–MS ESI (−)	–	–	5.4	−29.5	0.035	–
Oleic acid	282.2558	20.40	–	GC–MS/LC–MS ESI (−)	–	–	5.5	−28.9	0.039	–
Palmitic acid	256.2402	18.85	–	GC–MS/LC–MS ESI (−)	–	–	4.6	−20.7	0.016	–
Palmitoleic acid	254.2245	18.67	–	GC–MS/LC–MS ESI (−)	–	–	5.3	−33.9	0.017	–
Palmitoleoyl-EA	297.2667	21.15	–	LC–MS ESI (+)	1	[M+H]^+^	10.3	29.7	0.10	1.1
Stearic acid	284.2715	20.68	–	GC–MS/LC–MS ESI (−)	–	–	10.3	−17.4	0.0056	–
Glycerolipids										
MG(18:2)	354.2770	25.65	–	LC–MS ESI (+)	7	[M+H]^+^	23.3	36.1	0.024	1.4
Glycerophospholipids										
LPC(16:1)	493.3168	17.38	–	LC–MS ESI (+)	1	[M+H]^+^	9.3	21.1	0.054	1.2
LPC(17:1)	507.3316	18.22	–	LC–MS ESI (+)	2	[M+H]^+^	6.4	16.4	0.086	1.2
LPC(P-18:0)	507.3689	21.05	–	LC–MS ESI (+)	1	[M+H]^+^	4.2	14.6	0.10	1.2
LPC(22:6)	613.3371	17.71	–	LC–MS ESI (−)	1	[M+FA–H]^−^	4.1	15.8	0.049	–
PC(28:2)	709.4338	31.03	–	LC–MS ESI (−)	16	[M+Cl]^−^	6.6	−32.5	0.033	–
PC(36:5)	779.5465	31.18	–	LC–MS ESI (+)	1	[M+H]^+^	23.8	−31.2	0.014	1.2
Hydroxy acids and derivatives										
β-Hydroxybutyric acid	104.0473	8.25	–	GC–MS	–	–	5.3	−51.3	0.0079	–
Indoles and derivatives										
Serotonin	176.0949	13.21	0.76	CE–MS	2	[M+H]^+^	5.9	−43.9	0.15	–
Monocarboxylic acid amide										
Lactamide	89.0477	8.24	–	GC–MS	–	–	5.5	−29.1	0.0079	–
Organooxygen compounds										
Threitol	122.0579	12.89	–	GC–MS	–	–	5.4	−17.2	0.040	–
Pyrimidine nucleotides										
dUMP	308.0409	29.84	1.73	CE–MS	7	[M+H]^+^	12.7	31.6	0.051	–
Sphingolipids										
3-Ketosphingosine	297.2667	20.61	–	LC–MS ESI (+)	1	[M+H]^+^	17.5	35.2	0.063	1.2
PE-Cer(d30:2)	602.4423	30.54	–	LC–MS ESI (−)	10	[M−H]^−^	7.2	−22.7	0.042	–
Steroids and steroid derivatives										
Tetrahydroaldosterone-3- glucuronide	540.2570	1.08	–	LC–MS ESI (−)	3	[M−H]^−^	5.1	20.3	0.042	–
Multiple candidates										
Bile acid 1	374.2825	14.78	–	LC–MS ESI (+)	1	[M+H−H_2_O]^+^	9.7	57.6	0.032	1.3
Bile acid 2	396.2884	22.36	–	LC–MS ESI (+)	2	[M−H]^−^	11.6	219.1	0.041	1.0
Vitamine D2/5-Dehydroepisterol	379.3289	23.95	–	LC–MS ESI (+)	18	[M+H−H_2_O]^+^	5	15.4	1.62 × 10^−7^	2.7
Vitamine D3 metabolite	446.3387	30.48	–	LC–MS ESI (−)	1	[M−H]^−^	14.4	52.2	0.0063	–
Unknowns										
Unknown 1	445.1517	27.06	–	LC–MS ESI (−)	–	–	4.7	−26.3	0.026	–
Unknown 2	554.2056	31.03	–	LC–MS ESI (−)	–	–	8.3	−25.6	0.047	–
Unknown 3	563.4327	31.27	–	LC–MS ESI (+)	–	–	10.7	27.1	0.037	1.4

*RT* retention time expressed in minutes, *relative MT (RMT)* relative migration time based on the migration time of the internal standard added to each sample, *CV* coefficient of variation calculated in the QCs (30%), *% change* percentage of change calculated between pre-PD and controls, the sign (−) indicates that this metabolite is less abundant in pre-PD than in controls and the sign (+) indicates that this metabolite is more abundant in pre-PD than in the controls, *p*-value obtained with the *t*‐test after FDR correction, *VIP* variable of importance in projection.

Italic *p*-values are closed to be statistically significant.

### Metabolic alterations detected in plasma samples of pre-PD subjects

**Fig. 4:**
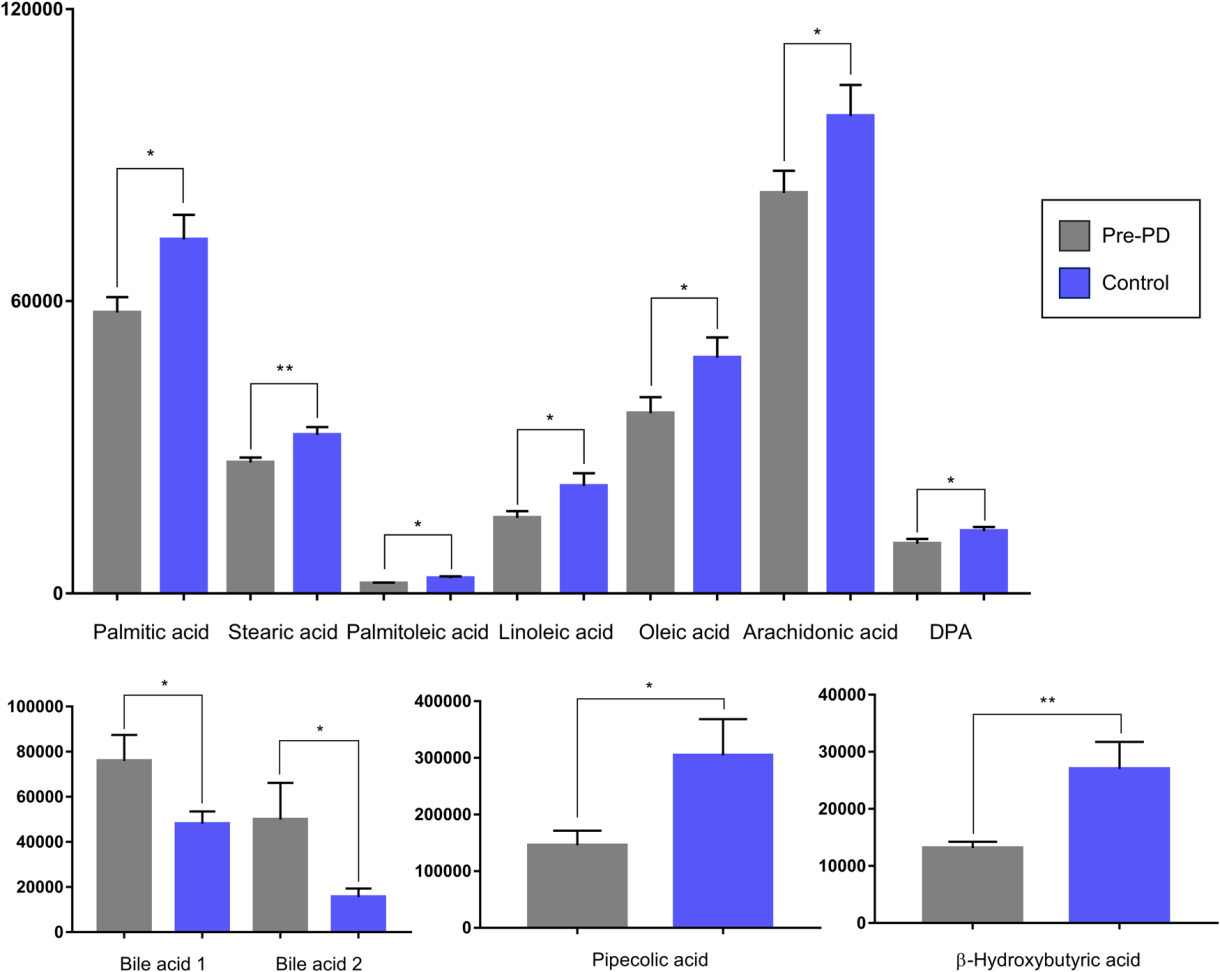
Abundance of the statistically significant FFAs, bile acids, pipecolic acid, and β-hydroxybutyric acid. The graphic reflects the differences between the pre-PD (grey bar) and the control (blue bar) group. The error bars represent the standard error of the mean (SEM). *p ≤ 0.05; **p ≤ 0.001.

**Fig. 5:**
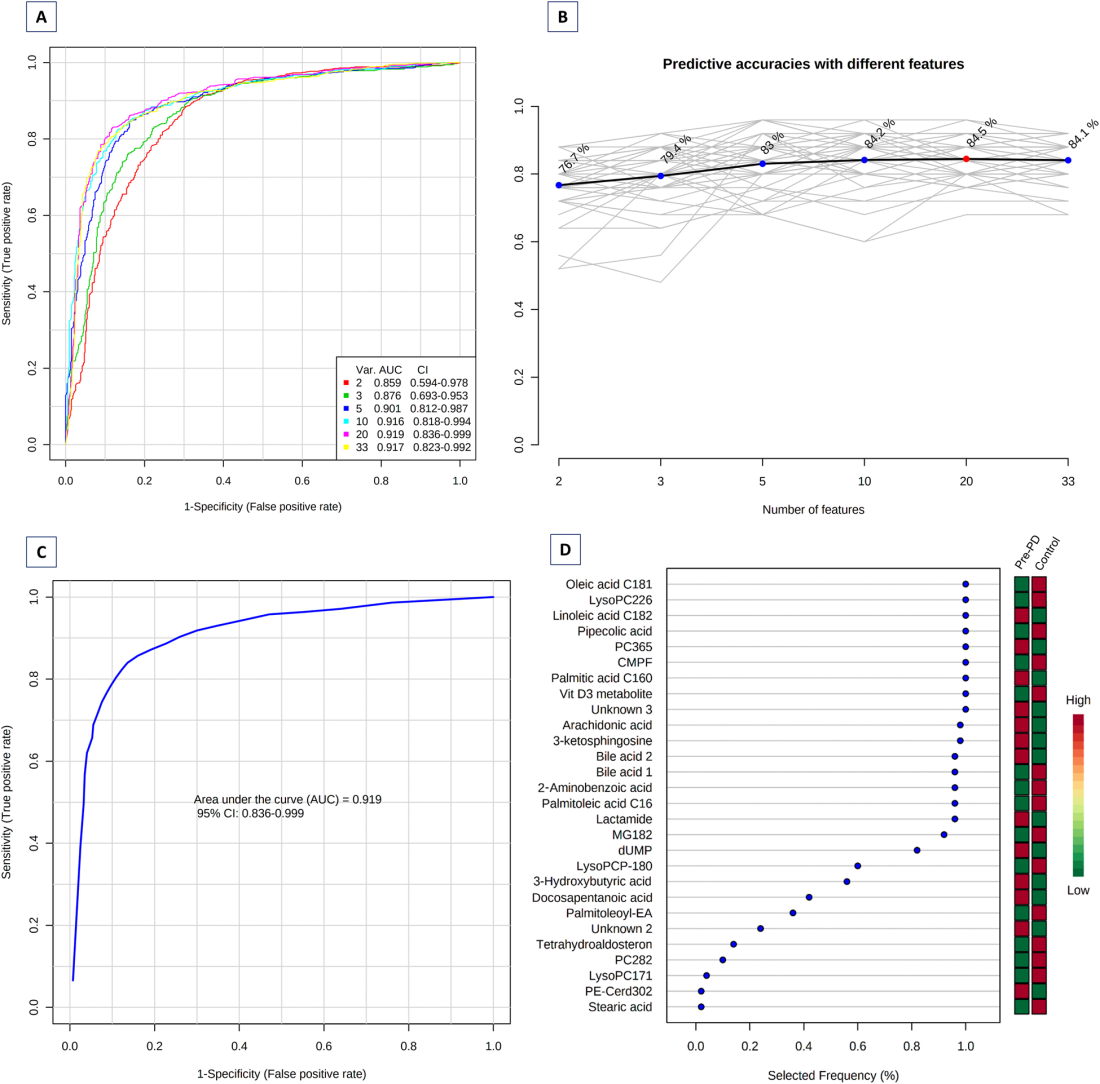
Biomarker prediction by Multivariate ROC curve based exploratory analysis. **A** Overview of all ROC curves created by MetaboAnalyst 4.0 from 6 different biomarker models considering different number of features (2, 3, 5, 10, 20, and 33) with their corresponding AUC value and confidence interval. **B** Graphic presenting the predictive accuracies of 6 different biomarker models. The red dot specifies the highest accuracy for the 20-feature panel of model 5. **C** ROC curve for selected biomarker model 5. **D** Top 28 potential biomarkers predicted based on their frequencies of being selected during cross validation.

Most of the identified signals in positive ionization belong to the class of lipids, mainly lysophosphatidylcholines (LPC), while in negative ionization mode most common metabolites were saturated, monounsaturated, and polyunsaturated fatty acids. The levels of LPC(16:1), LPC(17:1), LPC(P-18:0), and LPC(22:6) were increased, while phosphatidylcholines PC(36:5) and PC(28:2) were significantly decreased in pre-PD samples compared to healthy controls. Additionally, the bile acid levels were upregulated in pre-PD samples (Fig. 4). However, the type of bile acid was not established according to the spectra and it was only identified according to the compound group. The Palmitoleoyl ethanolamide, MG(18:2), 3-ketosphingosine, and vitamin D2/5-dehydroepisterol were also increased in plasma samples of pre-PD subjects. Regarding the LC–MS analysis in negative ionization mode, the arachidonic acid, docosapentaenoic acid, linoleic acid, oleic acid, stearic, palmitic acid, and palmitoleic acid were decreased in plasma samples of pre-PD subjects compared with healthy controls, same as levels of tetrahydroaldosterone-3-glucuronide and 3-carboxy-4-methyl-5-propyl-2-furanpropanoic acid. As well as after LC–MS analysis, most of the significantly affected compounds obtained after GC–MS analysis were also identified as FFAs. Palmitoleic, linoleic, oleic, palmitic, and stearic acids were significantly decreased in Pre-PD plasma samples (Table 1, Fig. 4). Furthermore, the levels of β‐hydroxybutyric acid, 2‐hydroxy‐3‐methylbutyric acid, lactamide, and threitol were also reduced in plasma samples of pre-PD subjects compared with healthy controls. The detection of these changes in the FFAs levels by two independent analytical platforms not only increases the annotations’ confidence but also reinforces the robustness of biological observations. The pipecolic acid and 2‐aminobenzoic acid levels detected by CE–MS were decreased, while the 3‐methoxytyrosin showed an increment in the plasma samples of pre-PD group. Finally, the potential biomarkers obtained from the multivariate ROC curve exploration module, oleic acid, LPC(22:6), linoleic acid, pipecolic acid, PC(36:5), CMPF, palmitic acid, vitamin D3 metabolite, arachidonic acid, 3-ketosphingosine, bile acids, 2-aminobenzoic acid, palmitoleic acid, lactamide, MG(18:2), and dUMP, presented a selection frequency between 0.8 and 1.0 (i.e., selected between 80 and 100% of the time in the model) of the SVM feature selection algorithm (Fig. 5).

### Free fatty acid-targeted analysis for biological validation

**Fig. 6:**
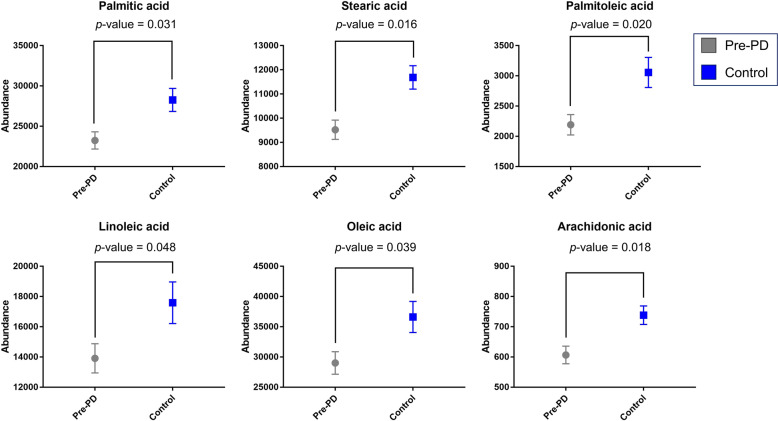
Abundance of the statistically significant FFAs after HPLC-QqQ-MS analysis. The graphic reflects the differences between the pre-PD (grey bar) and the control (blue bar) group. The error bars represent the standard error of the mean (SEM). The *p*-values displayed are the corrected *p*-values after Benjamini-Hochberg FDR correction test.

After obtaining the palmitic acid, stearic acid, palmitoleic acid, oleic acid, linoleic acid, and arachidonic acid as statistically significant by LC–MS and GC–MS analyses, and based on the Multivariate ROC curve analysis results, the plasma samples (Pre-PD *n* = 39; control *n* = 39) were measured by HPLC-ESI-QqQ-MS method operating in MRM in ESI(−) mode attempting to confirm these new metabolic findings. The plasma samples preparation, the analytical conditions, and the data processing of the targeted analysis are described in Supplementary Note 1. The calculated *t*-test *p*-values corrected by Benjamini–Hochberg FDR correction test (MATLAB R2015a software, Mathworks, Inc., Natick, USA) for the six FFAs are displayed in Fig. 6. As it can be appreciated in the graphic, the six lipid species were also downregulated in the Pre-PD group when comparing with the control group. According to the results, these markers are highly significant and, thus, corroborate that these six FFAs detected using a targeted approach were in full agreement with our original data from untargeted analyses.

## Discussion

Parkinson’s disease is a progressive, complex, multisystem neurodegenerative disorder, characterized by typical movement symptoms^22,23^ and cognitive impairments that affect usual functioning. Metabolomics as a comprehensive technique might provide insight into altered endogenous, but also exogenous metabolites^13^. Due to complexity and heterogeneity, several metabolic pathways are involved in its development and pathogenesis^13^. It is crucial to enlighten that metabolites were already altered before PD symptoms are developed because the sampling has been performed before the development of the disease, while subjects still did not have any symptoms. Several metabolites, part of fatty acid and dicarboxylic acid metabolisms were changed in subjects with pre-PD compared with control subjects who did not develop the disease.

**Fig. 7:**
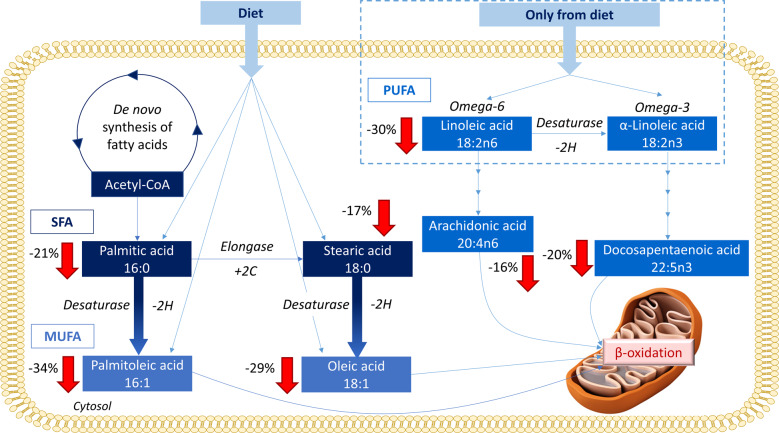
Free fatty acids metabolism and their trends observed in this study when compared the pre-PD group with healthy subjects. The arrows represent the trend of each metabolite within the comparison, displaying the percentages of change of each of them next to the arrows.

In this study, decreased levels of multiple fatty acyls including saturated fatty acids (SFA), monounsaturated fatty acids (MUFA), and polyunsaturated fatty acids (PUFA) were noticed in pre-symptomatic PD subjects (Fig. 7). This is in agreement with recent studies that recognized a remarkable reduction of the levels of several FFAs in PD patients^10,20,24^.

Fatty acids are vital for a correct brain function since it depends on dietary fatty acid intake^25^. Lipids and fatty acid dyshomeostasis is associated with many neurodegenerative disorders, neuroinflammation, and oxidative stress^15^, same as with apoptotic signaling and mitochondrial dysfunction^10,13,14^. We found lower levels of the SFA palmitic acid (16:0) and stearic acid (18:0) in the plasma of pre-PD subjects^26^. Currently, it seems that there is no direct connection between the SFA intake and PD risk in humans^27^. Interestingly, higher levels of both SFA have been observed in lipid rafts from the frontal cortex of PD patients compared to healthy controls^28^. Moreover, the α-synuclein modulates the uptake of palmitic acid into the brain^29^. Therefore, the accumulation of this protein in PD brains might lead to increased levels of palmitic acid, which in turn can trigger some of its neuropathological activities. Consequently, it can be hypothesized that the lower levels of both SFA in plasma of the pre-PD subjects might be due to an early and progressive migration and reuptake of these fatty acids to the brain. Regarding the MUFA, palmitoleic acid (16:1) and oleic acid (18:1) are recognized of great importance for human metabolism since they are considered anti-inflammatory compounds with neuronal protective effects. Palmitoleic acid is one of the principal components of the human adipose tissue, muscle, and liver together with palmitic, stearic, oleic, and linoleic acids, whose levels have been also detected as significantly reduced in the PD group. Oleic acid aids in the synthesis of hormones, and it is present in all tissues, generally found in higher concentrations in the intestine and liver. Both MUFAs can be accumulated in adipose tissue as triacylglycerols, cholesteryl esters, or phospholipids, and they participate in lipid transport and metabolism^30^. Moreover, they are essential players in cellular signaling and lipid peroxidation^31,32^. Interestingly, a recent study has demonstrated that oleic acid presents detrimental effects in the α-synuclein homeostasis since it induces α-synuclein inclusion formation in human neural cells^33^. Therefore, the lower levels of oleic acid in plasma might be also due to its early reuptake by the brain. Regarding the PUFAs, decreased levels of arachidonic, docosapentaenoic, and linoleic acids were found in the Pre-PD group when compared to the control group. Although the epidemiological evidence suggesting that dietary fat consumption may be associated with PD risk is not consistent, a significant number of PD animal models and epidemiological research studies have proved that PUFAs might play many critical roles in this regard. The cell membrane sequencing, gene transcription, cell signal transduction, and protease activation of glial and neuronal cells may influence the PD progress^34^. The elevated PD risk may result from the dietary fat effects, including increased oxidative stress and neuroinflammation, which can potentially worsen the dopaminergic neuron loss due to neurotoxicity.

Although the SFAs decreased levels observed in the pre-PD group is the opposite to the observations recently reported in a 6-OHDA rat model of PD^35^, a recent study conducted with plasma samples collected from PD patients’ uncovered diminished levels of 4 SFAs, including the stearic acid (18:0) in the PD group compared with their corresponding control group^20^. The study also revealed reduced levels of 4 MFA, including the oleic acid (18:1), and 9 PUFA, including the linoleic acid (18:2), arachidonic acid (20:4), and DPA (22:5)^20^. Therefore, thanks to the analysis of the unique pre-symptomatic cohort that our study is based on, it can be stated that the metabolic remodelings observed on the FFAs levels begin many years before the PD onset.

The common feature among neurodegenerative disorders such as PD or AD is the lack of robust diagnostics and prognostic biomarkers. The study conducted by Goozee et al. (Alterations in erythrocyte fatty acid composition in preclinical Alzheimer’s disease) with preclinical AD subjects with higher β-amyloid load unveiled elevated levels of AA, oleic acid, and stearic acid and decreased levels of DHA in erythrocyte samples. Therefore, FFAs levels in blood samples could be considered as potential discriminatory markers between AD and PD future development.

This study also unveiled decreased levels of pipecolic acid among pre-PD subjects. It is becoming increasingly evident that intestinal microbiota influences gut–brain communication. Indeed, the disruption of the gut–brain axis has been shown to be involved in the pathogenesis of a broad range of diseases, including Parkinson’s disease^36^. The premise that PD development is triggered following continuous gut aggravation has gathered significant strength recently. In fact, the enteric αSyn has been associated with greater intestinal permeability^37^. Moreover, it exists a positive relationship between inflammatory bowel diseases and future PD risk in various populations^38^. A previous study demonstrated how metabolites involved in the correct brain function were influenced by the intestinal microbiota, including the pipecolic acid^39^. This metabolite comes from the degradation of the amino acid lysine in the cerebral peroxisomes, which are multifunctional organelles involved in many roles, including ROS metabolism, fatty acid oxidation, ether lipid synthesis, bile acid synthesis, and cholesterol transport. Peroxisomal dysfunction has been linked to neurodegenerative disorders, including Parkinson’s disease (PD)^40^. It has been previously reported that pipecolic acid can act as a neurotransmitter modulating the gamma-aminobutyric acid (GABA)ergic transmission^41^. Furthermore, pipecolic acid can be taken up by cerebral mitochondria, the principal responsible for cellular apoptosis activation, stimulating neuronal cell death^41^. Therefore, altered levels of pipecolic acid might be indicative of the initial stages of the gut–brain axis dysregulation and a peroxisomal failure. In addition to pipecolic acid, we did not observe any other significantly changed amino acid, while other articles reported altered levels of certain amino acids in PD subjects. Increased levels of valine, alanine, leucine, isoleucine, methionine, threonine, and serine were reported in PD patients^10,13,42^. However, as our study samples were collected prior to disease development, changes in the amino acid levels might be considered metabolic biomarkers of advanced stages of the PD.

We found β-Hydroxybutyric acid levels to be decreased (−51%) in people that developed PD. This metabolite is a ketone body synthesized in liver mitochondria from acetyl-CoA and serves as an alternative fuel source for extrahepatic tissues including the brain. It is a product of healthy metabolism of fatty acid oxidation together with acetoacetate. It has been previously described that the administration of β-Hydroxybutyric acid protects both mesencephalic and dopaminergic neurons from MPP^+^ and MPTP toxicity, respectively^43,44^. Moreover, patients able to tolerate a ketogenic diet improved their Unified Parkinson Disease Rating Scale scores with no harmful effects^45^. Consequently, a decrease in the β-Hydroxybutyric acid levels might indicate the initial stage of the mitochondrial impairment that plays a central role in the PD pathophysiology.

We have detected an increase in 3-carboxy-4-methyl-5-propyl-2-furanpropionic acid (CMPF) of a 32% in pre-PD plasma samples. CMPF, a metabolite of furan fatty acids, is a potent uremic toxin found accumulated in the blood, CSF, and brain tissue of uremic patients and has been associated with impaired glucose tolerance, type 2 diabetes (T2D), and progression of pre-diabetes to T2D^46^. Furthermore, it has been demonstrated that CMPF undergoes efflux across the BBB, which might help understand the mechanisms behind the development of neurological symptoms of the uremic syndrome in patients with chronic renal failure^47^. Recently, a large-scale Asian study carried out by Nam et al.^48^ described how chronic renal dysfunction is positively associated with an increased risk of PD incidence. Consequently, the increase in CMPF levels may suggest the onset of latent renal failure and an increased risk of developing PD in the future.

In this study, increased levels of bile acids have also been observed. They were identified only up to subclass level; therefore, altered levels of precise bile acids have not been reported. However, it is known that bile acids are involved and changed in subjects with PD. In fact, in accordance with our results, it has been recently described an increment of the levels of bile acids in plasma samples of PD patients^20^. Consequently, elevations of bile acid levels can also be detected many years before the PD development. Due to their role, it is assumed that the downregulation of fatty acids and phospholipids and other perturbations in lipid metabolism might be associated with impaired bile acid metabolism. Consequently, it could cause further disturbances in energy production^49^. As mentioned, energy production might be altered in PD subjects. In association with fatty acid metabolism, the TCA cycle plays an important role in energy metabolism. Dysregulation of the TCA cycle is possibly associated with PD progression and α-synuclein pathology, while fatty acid metabolism might be associated with α-synuclein aggregation^13^. We identified decreased levels of cis-aconitic acid in subjects with PD, compared with healthy control subjects. Several studies showed decreased levels of TCA metabolites in postmortem brain or cell cultures. It is assumed that the TCA cycle has an important role as part of energy metabolism in the degeneration of dopaminergic cells through complex gene × environment interaction^13^. At the beginning of neurodegenerative processes in PD, TCA cycle is downregulated due to mitochondrial dysfunction or energy deficient^13^, which is in correspondence with the downregulation of fatty acid metabolites also due to disturbances in mitochondrial function and energy production^49^. Although the difference between pre-PD subjects and controls was not significant in our study, it has been newly uncovered diminished cis-aconitic acid levels in PD patients^20^. Therefore, reduced levels of TCA cycle metabolites might be related to the future onset of neurodegeneration, which might have been shown in the decreased level of cis-aconitic acid observed in this study among subjects that developed PD.

The metabolism of tryptophan and its co-metabolites are associated with PD progression and development^11,13^. Postmortem examinations in PD patients showed decreased levels of metabolites that take part in tryptophan metabolism, the same as altered tryptophan levels in cerebrospinal fluid and plasma. It is assumed that dysregulation of this metabolism leads to neurotoxicity^50^, which might act as a trigger for PD development. This study has shown decreased levels of tryptophan co-metabolites, such as 2-aminobenzoic acid and serotonin in PD patients. Serotonin is a neurotransmitter produced from amino acid tryptophan, where its reduction might be a result of tryptophan dysregulation, however, it did not differ significantly between PD subjects and healthy control subjects. Unlike serotonin, 2-aminobenzoic acid was significantly decreased in PD subjects, which might confirm the possible association of altered tryptophan metabolism in the early stage of disease development. Other amino acids or metabolites part of purine or dopamine metabolism have not been observed in this study, probably such alterations might still not happen in subjects that were healthy at the time of sample collection.

Our study’s strengths include a unique cohort composed of participants from the European Prospective Study on Nutrition and Cancer (EPIC) followed for almost 15 years. The fact that none of the participants presented PD or disease-related symptoms at the sample collection time makes this study of exceptional value for discovering potential biomarkers for the early diagnosis of this devastating disease. The multiplatform approach adopted for this untargeted high-resolution metabolomics study shed light, for the first time, on the metabolic remodeling taking place many years before PD development in 33 plasma metabolites. Among those changes, several FFAs were detected as significantly affected by two independent analytical platforms (GC–MS and LC–QTOF–MS), and subsequently validated by HPLC-QqQ-MS, increasing the annotations’ confidence, and reinforcing the robustness of biological observations. The study’s limitations include modest sample size, bestowing this work the category of a pilot study. Additionally, our cohort consists of subjects living in Spain, and, therefore, our findings may not be generalizable to other geographical areas. However, our results open the door for future studies with more significant independent cohorts to replicate and validate such metabolites to consider them as prognostic biomarkers of PD development.

Through a multiplatform untargeted metabolomics-based approach we have identified for the first time a total of 33 altered metabolites in plasma samples from subjects that did not present any pathology when the samples were collected and, many years later, they developed PD. Several pieces of evidence indicate the implication of mitochondrial dysfunction and oxidative stress, which is reflected in the metabolite changes of PD pathogenesis. According to our data, it is evident that changes in fatty acid metabolism and their corresponding metabolic pathways are altered long before PD’s first symptoms are observed. Likewise, in our study, these metabolites levels were significantly reduced compared to subjects that did not develop PD during the time they were followed up. The alteration in the levels of palmitic, stearic, palmitoleic, oleic, linoleic, and arachidonic acids were then validated adopting a targeted HPLC-QqQ-MS metabolomics strategy unveiling the great significance of these metabolic markers for the potential early detection of the PD. Our results are mostly in agreement with recent metabolomics-based findings where the levels of 17 FFAs were found decreased in plasma samples collected from PD patients, including the stearic, oleic, linoleic, arachidonic, and docosapentaenoic acids. In the same line, increased levels of bile acids and decreased cis-aconitic acid levels were also found in PD patients, highlighting that these metabolite modifications begin many years before the PD development and are maintained when suffering this disease. Furthermore, the detected alteration in pipecolic acid levels lead to hypothesize about the early triggering of the gut–brain axis dysregulation and peroxisomal failure. Although the biological purpose of these events is still unknown, the mechanisms involved in the suggested mitochondrial dysfunction, oxidative stress, and gut–brain axis dysregulation seem to appear long before the development of this disease. Therefore, the remodeled metabolic pathways highlighted in this study might be considered as worthy potential markers whose alteration might lead to the development of PD hallmarks in the future.

## Methods

### Study population

The European Prospective Investigation into Cancer and Nutrition is an ongoing multicenter prospective cohort study designed to investigate the relationship between diet, nutrition, and metabolic factors with cancer. Descriptions of study design, population, and baseline data collection of the cohort have been reported in detail previously^51^.

The Spanish EPIC cohort consists of 41,437 participants (62% women), aged 29–69 years, enrolled in five Spanish regions: three in the North (Asturias, Gipuzkoa, and Navarra) and two in the South (Granada and Murcia) between 1993 and 1996. The participants were healthy volunteers, mostly blood donors (67%) but also employees from private companies (3%), civil servants (5%), and the general population (23%) all of whom were fully covered by the public health system. The exclusion criteria were pregnancy, lactation, and not being physically or mentally capable of participating^52^.

The EPIC-Spain Parkinson Cohort study comprises the three centers available on Parkinson incident data Gipuzkoa, Navarra, and Murcia. The study sample consisted of 25,015 participants (57% women), aged 30–70 years at recruitment. This study was approved by the Ethics Committee for clinical research of the Basque Country PI2017031 and written informed consent was obtained from pre-PD subjects and controls according to approved protocols.

### Lifestyle, diet, anthropometry, and clinical data

Baseline dietary and lifestyle data were collected in face-to-face interviews using validated questionnaires. Detailed descriptions of both the dietary and lifestyle questionnaires used have been published previously. A lifestyle questionnaire was used to collect information on sociodemographic characteristics, lifestyle, and medical history as well as on reproductive factors in women. Anthropometric measures, height, weight, and waist circumference were measured in all of the participants using standard procedures. Body mass index was computed as weight (in kilograms) divided by height (in meters) squared^51,53^.

### Case ascertainment

Potential cases were identified using different sources of information depending on the center. Each center used at least two of the following: (1) record linkage with Primary Health records using whether the ICD-9 codes 332 or the International Classification of Primary Care (ICPC) codes N87 for Parkinson’s disease; (2) record linkage with Prescriptions registry including subjects with at least one prescription of any of the N04–Antiparkinson Drugs of the ATC/DDD index (N04—Antiparkinson Drugs; N04A—Anticholinergic agents; N04B—Dopaminergic agents); (3) record linkage with mortality registry using the ICD-9 codes 332 for PD; (4) hospital discharge database using the ICD-9 codes 332 for PD; (5) death certificates using the ICD-10 code G20. Subjects identified in each of these sources were linked on EPIC identifier and duplicates were discarded.

After a follow-up period running from recruitment to June 2011, about 90 individuals developed PD. Each diagnosis was based on a matrix combining two variables: the amount and quality of data available, and the degree of confidence of the neurologist expert in movement disorders reviewing the evidence. Diagnoses were defined as “definite” only when the degree of confidence of the neurologist was high and data quality excellent; diagnoses were defined as “very likely” in case the degree of confidence of the neurologist was high, but data quality was either good or poor; and defined as “probable” when the degree of confidence of the neurologist was medium and data quality was either excellent or good; finally, diagnoses were defined as “possible” in all remaining cases. The centers of Navarra and Murcia were able to verify all potential cases and San Sebastian could verify up to 84.8% of potential cases.

The vital status of the cohort and cause of death were assessed through record linkage with the regional mortality registry and the Spanish National Statistics Institute (www.ine.es).

### Design of nested case–control study

For each case subject up to two control subjects were randomly selected among appropriate risk sets consisting of all-female cohort members with a blood sample, alive, and free of cancer at the time of diagnosis of the index case. An incidence density sampling protocol was used, such that, in principle, control subjects could include study participants who became a case later in time and each control subject could be sampled more than once—the control subjects are actually drawn, however, did not include any of the future cases of ovarian cancer detected so far in the EPIC cohort. Case and control subjects were matched on study recruitment center, age at blood donation (±6 months), time of the day of blood collection (±1 h), fasting status (<3 h, 3–6 h, >6 h), follow-up time, and menopausal status at blood collection (premenopausal, perimenopausal, postmenopausal), current use of exogenous hormones (oral contraceptives, HRT) at the time of blood draw, as well as menstrual cycle phase for premenopausal women (3–5 categories, depending on available data). Cases missing data on the phase of the menstrual cycle were matched to control subjects whose information on the menstrual cycle phase was also missing.

For this multiplatform untargeted metabolomics-based study, 39 individuals that developed PD (pre-PD group) and the corresponding control group were randomly selected, consisted both groups of 46% women, aged between 41 and 69 years old, and 54% males aged between 41 and 69 years old.

### Multiplatform untargeted metabolomics analysis

On the day of the interview, subjects were appointed for blood sample analysis within the next week at the primary care center, where the interview took place, and a 30 mL blood sample was obtained at baseline. Fasting venous blood samples were drawn and immediately processed and were divided into 0.5 mL aliquots of plasma, serum, concentrated red blood cells, and buffy coat, and stored in liquid nitrogen tanks at –190 °C until analysis. Plasma metabolites extraction was carried out according to previously reported standard protocols^54–56^. Briefly, for GC–MS analysis, protein precipitation was achieved by mixing 1 volume of plasma with 3 volumes of cold (−20 °C) acetonitrile, followed by methoximation with *O*-methoxyamine hydrochloride (15 mg/mL) in pyridine, and silylation with BSTFA:TMSC (99:1). Finally, 20 ppm of tricosane in heptane was added as an internal standard (IS). For CE–MS analysis, 100 µL of plasma was mixed with 100 µL of 0.2 M formic acid containing 5% acetonitrile and 0.4 mM methionine sulfone as IS. The sample was transferred to a centrifree ultracentrifugation device (Millipore Ireland Ltd., Carrigtohill, Ireland) with a 30 kDa protein cutoff for deproteinization through centrifugation (2000×*g*, 4 °C, 70 min). For LC–MS analysis, 100 µL of plasma was mixed with 300 µL of a cold mixture (−20 °C) of methanol:ethanol (1:1, v/v) for deproteinization. Samples were centrifuged (13,000×*g*, 4 °C, 20 min). After centrifugation, 100 µL of the supernatant was directly injected into the system. The detailed version of the sample treatment protocols, the reagents, solvents and standards used for the sample treatment and subsequent analyses, and the analytical setup for the LC–MS, GC–MS, and CE–MS analysis are described in Supplementary Note 1. Quality control samples (QC) were prepared by pooling and mixing equal volumes of each plasma sample to check the performance of the systems and the reproducibility of the sample treatment. Then, samples were randomized, and QCs were injected at the beginning, along the sequence, and at the end of the batch. Finally, two blank solutions were prepared along with the rest of the samples and analyzed at the beginning and at the end of the analytical sequence^57^.

### Data treatment after LC–MS and CE–MS analysis

The raw data obtained after the LC–MS and CE–MS analysis were processed using Agilent Technologies MassHunter Profinder B.08.00 SP1 software (Waldbronn, Germany) to clean the background noise and unrelated ions using the tool called Molecular Feature Extraction (MFE) included in the software in order to obtain a structured data matrix and appropriate format. This algorithm aligns all ions across the samples using mass and retention time (RT) to create a single spectrum for each group of compounds, allowing the next step of the analysis known as recursive feature extraction (RFE). The RFE integrates MFE and then uses the abundance of the molecule, mass, and RT of the previous results to improve the quality of the list of compounds, eliminating non-specific information and extracting the most important signals. Missing values were imputed using the K-nearest neighbors (kNN) algorithm^58^. Afterward, the data matrix was filtered by CV, maintaining those signals that, in the QCs, they presented a coefficient of variation (CV) below 30%. The filtered data matrix was imported into SIMCA P + 15 to generate a PCA and thus observe the trend of the QCs, detect possible outliers, and look for natural trends of the samples. The obtained PCA plot revealed an intra-batch effect due to gradual changes in the instrumental response that are often unavoidable, especially for long analytical sequences. In order to minimize the instrumental variation observed that hinders the power to detect the biological variation, the data were normalized by applying a correction called “quality control samples and support vector regression (QC-SVRC)”^59^. This SVR strategy uses a radial basis function kernel to correct the instrumental drift within a batch using data acquired from QC samples. Then, the normalized data matrix was filtered and the PCA plot was built. Finally, the data matrix was analyzed using univariate data analysis using MATLAB R2015a software (Mathworks, Inc., Natick, USA), applying a *t*-test to obtain statistically significant signals by comparing both groups (case vs. control) (*p*‐value <0.05). A standard Benjamini–Hochberg method was applied to control the false discovery rate (FDR) for multiple hypothesis testing. Finally, the percentage of change was calculated by comparing case vs. control. The metabolites that turned out to be statistically significant (*p*-value < 0.05) and showed % change >20% were tentatively annotated. Initially, the *m*/*z* of the significant metabolites was searched against multiple databases available online, including METLIN (http://metlin.scripps.edu), LipidsMAPS (http:// lipidMAPS.org) and KEGG (http://www.genome.jp/kegg/), all of which have been joined into an “in‐house” developed search engine, CEU MassMediator (http://ceumass.eps.uspceu.es/)^60,61^. Aiming to obtain additional information for some identities, HMDB (http://hmdb.ca) was also consulted. Features that were tentatively assigned to metabolites from the databases were based on: (1) mass accuracy (maximum error mass 20 ppm), (2) isotopic pattern distribution, (3) possibility of cation and anion formation, (4) adduct formation, and (5) elution order of the compounds based on the chromatographic conditions. Additionally, an “in-house” CE–MS library built with authentic standards was used to compare the relative migration time (RMT) of the significantly affected metabolites to increase the confidence of the annotations. As it can be observed in Table 1, some metabolites were detected in at least two techniques presenting the same trend. This fact also ensures the great analytical performance and reproducibility of the three analytical platforms, increases the confidence level of the metabolite annotations, and verifies the consistency of the results throughout the whole study. Finally, a supervised OPLS-DA model was obtained for the LC–MS ESI(+) data^21^. The metabolites presenting variable importance in projection (VIP) ≥1 and jackknifing confidence interval, not including the zero value were selected as statistically significant from the OPLS‐DA model. Finally, validation of the model was performed with cross-validation and CV-ANOVA tool provided by SIMPA-P+ software.

### Data treatment after GC–MS analysis

The chromatograms obtained from each of the plasma samples, the QCs, and the internal standard (IS) signal were visually examined to ensure the quality of the obtained profiles and the reproducibility of the IS signal using Agilent MassHunter Qualitative B.08.00 software. Deconvolution and metabolite identification was achieved using the Agilent MassHunter Unknowns Analysis Tool 9.0. The software assigned a chemical identity to each of the signals obtained after the search in two commercial libraries: the Fiehn library version 2013, and the NIST library version 2017. The identities were assigned according to the retention time (RT) and spectra extracted during deconvolution when the software compared them with each compound included in the libraries. Next, the obtained data were aligned using the MassProfiler Professional B.12.1 software (Agilent Technologies) and exported to Agilent MassHunter Quantitative Analysis version B.09.00 to assign the main ions and the integration of each of the signals. As in the LC–MS and CE–MS analysis, the missing values were estimated using the kNN (k-nearest neighbors) algorithm^58^. Experimental and analytical variations were excluded by performing normalization. Firstly, normalization was tested for the abundance of the internal standard. The quality of the data was assured by maintaining only those signals presenting a CV below 30 % in QC samples. The resulted data matrix was imported in SIMCA P + 15.0 to generate the PCA plot. This non-supervised model also showed the presence of an intra-batch effect in the data obtained by this technique. Therefore, the data matrix was normalized by applying the QC-SVRC correction, filtered by CV in the QCs, and the PCA plot was generated^59^. Finally, the data matrix was analyzed using univariate data analysis using MATLAB R2015a software (Mathworks, Inc., Natick, USA), applying a *t*-test (*p*‐value < 0.05). The false discovery rate at level *α* = 0.05 was controlled by the Benjamini−Hochberg correction test.

### Identification of potential metabolite biomarkers

For biomarker prediction, the Multivariate ROC plot-based exploratory analysis (Explorer) was performed in MetaboAnalyst 4.0 (https://www.metaboanalyst.ca/) (Fig. 5A–D). This analysis performs automated important feature identification and performance evaluation. The ROC curve analyses were based on Linear Support Vector Machine (SVM), and the ROC plots were generated by Monte-Carlo cross-validation using balanced sub-sampling. In each cross-validation, two-thirds (2/3) of the samples are employed to evaluate the feature importance. The principal features (2, 3, 5, 10 …100 as maximum) are then exploited to construct the classification models, which are validated on the remaining 1/3 of the samples. The procedure is replicated multiple times to calculate the performance and the confidence interval of each model. Multiple algorithms are available for classification and feature ranking methods. For our data, the classification method selected was Linear SVM, and the feature ranking method selected was the SVM built-in algorithm^62^.

## Supplementary information

Supplementary Information

## Data Availability

The data sets used and/or analyzed during the current study are available at the Metabolomics Workbench platform (access no. ST001814; http://www.metabolomicsworkbench.org/).

## References

[1] Han W, Sapkota S, Camicioli R, Dixon RA, Li L (2017). Profiling novel metabolic biomarkers for Parkinson’s disease using in‐depth metabolomic analysis. Mov. Disord..

[2] De Virgilio A (2016). Parkinson’s disease: autoimmunity and neuroinflammation. Autoimmun. Rev..

[3] Sveinbjornsdottir S (2016). The clinical symptoms of Parkinson’s disease. J. Neurochem..

[4] Cookson, M. R. in *Disease-Modifying Targets in Neurodegenerative Disorders* Ch. 6 (ed. V. Baekelandt & E. Lobbestael) 157–174 (Academic Press, 2017).

[5] Pfeiffer RF (2016). Non-motor symptoms in Parkinson’s disease. Parkinsonism Relat. Disord..

[6] Blesa J, Trigo-Damas I, Quiroga-Varela A, Jackson-Lewis VR (2015). Oxidative stress and Parkinson’s disease. Front. Neuroanat..

[7] Bose A, Beal MF (2016). Mitochondrial dysfunction in Parkinson’s disease. J. Neurochem..

[8] Cheng HC, Ulane CM, Burke RE (2010). Clinical progression in Parkinson disease and the neurobiology of axons. Ann. Neurol..

[9] Wishart DS (2016). Emerging applications of metabolomics in drug discovery and precision medicine. Nat. Rev. Drug Discov..

[10] Havelund JF, Heegaard NH, Færgeman NJ, Gramsbergen JB (2017). Biomarker research in Parkinson’s disease using metabolite profiling. Metabolites.

[11] Luan H (2015). LC–MS-based urinary metabolite signatures in idiopathic Parkinson’s disease. J. Proteome Res..

[12] Luan H (2015). Comprehensive urinary metabolomic profiling and identification of potential noninvasive marker for idiopathic Parkinson’s disease. Sci. Rep..

[13] Shao Y, Le W (2019). Recent advances and perspectives of metabolomics-based investigations in Parkinson’s disease. Mol. Neurodegener..

[14] LeWitt PA, Li J, Lu M, Guo L, Auinger P (2017). Metabolomic biomarkers as strong correlates of Parkinson disease progression. Neurology.

[15] Willkommen, D. et al. Metabolomic investigations in cerebrospinal fluid of Parkinson’s disease. *PLoS ONE* **13**, e0208752 (2018). 10.1371/journal.pone.0208752 PMC6287824 30532185

[16] Trezzi JP (2017). Distinct metabolomic signature in cerebrospinal fluid in early Parkinson’s disease. Mov. Disord..

[17] Hatano T, Saiki S, Okuzumi A, Mohney RP, Hattori N (2016). Identification of novel biomarkers for Parkinson’s disease by metabolomic technologies. J. Neurol. Neurosurg. Psychiatry.

[18] LeWitt P (2013). Arizona Parkinson’s Disease Consortium. 5-Hydroxykynurenine and other biomarkers of Parkinson’s disease discovered by metabolomic analysis. Mov. Disord..

[19] Bogdanov M (2008). Metabolomic profiling to develop blood biomarkers for Parkinson’s disease. Brain.

[20] Shao Y (2021). Comprehensive metabolic profiling of Parkinson’s disease by liquid chromatography-mass spectrometry. Mol. Neurodegener..

[21] Godzien J, Ciborowski M, Angulo S, Barbas C (2013). From numbers to a biological sense: How the strategy chosen for metabolomics data treatment may affect final results. A practical example based on urine fingerprints obtained by LC-MS. Electrophoresis.

[22] Emamzadeh FN, Surguchov A (2018). Parkinson’s disease: biomarkers, treatment, and risk factors. Front. Neurosci..

[23] Kalia LV, Lang AE (2015). Parkinson’s disease. Lancet.

[24] Schmid SP (2012). Cerebrospinal fluid fatty acids in glucocerebrosidase‐associated Parkinson’s disease. Mov. Disord..

[25] Chang C-Y, Ke D-S, Chen J-Y (2009). Essential fatty acids and human brain. Acta Neurol. Taiwan.

[26] Xicoy H, Wieringa B, Martens GJ (2019). The role of lipids in Parkinson’s disease. Cells.

[27] Miyake Y (2010). Dietary fat intake and risk of Parkinson’s disease: a case-control study in Japan. J. neurological Sci..

[28] Fabelo, N. et al. Severe alterations in lipid composition of frontal cortex lipid rafts from Parkinson’s disease and incidental Parkinson’s. *Mol. Med.* **17**, 1107–1118 (2010). 10.2119/molmed.2011.00119 PMC3188884 21717034

[29] Golovko MY (2005). α-synuclein gene deletion decreases brain palmitate uptake and alters the palmitate metabolism in the absence of α-synuclein palmitate binding. Biochemistry.

[30] Frigolet ME, Gutiérrez-Aguilar R (2017). The role of the novel lipokine palmitoleic acid in health and disease. Adv. Nutr..

[31] Hodson L, Karpe F (2013). Is there something special about palmitoleate?. Curr. Opin. Clin. Nutr. Metab. Care.

[32] Carrillo Pérez, C., Cavia Camarero, M.d.M. & Alonso de la Torre, S. Role of oleic acid in immune system; mechanism of action; a review. *Nutr. Hosp.* **27**, 978–990 (2012). 10.3305/nh.2012.27.4.5783 23165533

[33] Fanning S (2019). Lipidomic analysis of α-synuclein neurotoxicity identifies stearoyl CoA desaturase as a target for Parkinson treatment. Mol. Cell.

[34] Qu Y, Chen X, Xu M-M, Sun Q (2019). Relationship between high dietary fat intake and Parkinson’s disease risk: a meta-analysis. Neural Regener. Res..

[35] Shah A, Han P, Wong M-Y, Chang RC-C, Legido-Quigley C (2019). Palmitate and stearate are increased in the plasma in a 6-OHDA model of Parkinson’s disease. Metabolites.

[36] Foster JA, Neufeld K-AM (2013). Gut–brain axis: how the microbiome influences anxiety and depression. Trends Neurosci..

[37] Forsyth, C. B. et al. Increased intestinal permeability correlates with sigmoid mucosa alpha-synuclein staining and endotoxin exposure markers in early Parkinson’s disease. *PLoS ONE* **6**, e28032 (2011). 10.1371/journal.pone.0028032 PMC3228722 22145021

[38] Gorecki AM (2019). Altered gut microbiome in Parkinson’s disease and the influence of lipopolysaccharide in a human α-synuclein over-expressing mouse model. Front. Neurosci..

[39] Matsumoto M (2013). Cerebral low-molecular metabolites influenced by intestinal microbiota: a pilot study. Front. Syst. Neurosci..

[40] Cipolla CM, Lodhi IJ (2017). Peroxisomal dysfunction in age-related diseases. Trends Endocrinol. Metab..

[41] Matsumoto S (2003). Pipecolic acid induces apoptosis in neuronal cells. Brain Res..

[42] Mally J, Szalai G, Stone T (1997). Changes in the concentration of amino acids in serum and cerebrospinal fluid of patients with Parkinson’s disease. J. Neurol. Sci..

[43] Kashiwaya Y (2000). d-β-Hydroxybutyrate protects neurons in models of Alzheimer’s and Parkinson’s disease. Proc. Natl Acad. Sci. USA.

[44] Tieu K (2003). D-β-Hydroxybutyrate rescues mitochondrial respiration and mitigates features of Parkinson disease. J. Clin. Investig..

[45] VanItallie TB (2005). Treatment of Parkinson disease with diet-induced hyperketonemia: a feasibility study. Neurology.

[46] Gordon-Dseagu VL (2019). The association of sleep with metabolic pathways and metabolites: evidence from the dietary approaches to stop hypertension (DASH)—sodium feeding study. Metabolomics.

[47] Deguchi T, Isozaki K, Yousuke K, Terasaki T, Otagiri M (2006). Involvement of organic anion transporters in the efflux of uremic toxins across the blood–brain barrier. J. Neurochemistry.

[48] Nam GE (2019). Chronic renal dysfunction, proteinuria, and risk of Parkinson’s disease in the elderly. Mov. Disord..

[49] Zhao H (2018). Potential biomarkers of Parkinson’s disease revealed by plasma metabolic profiling. J. Chromatogr. B.

[50] Szabó N, Kincses ZT, Toldi J, Vécsei L (2011). Altered tryptophan metabolism in Parkinson’s disease: a possible novel therapeutic approach. J. Neurol. Sci..

[51] Riboli E (2002). European Prospective Investigation into Cancer and Nutrition (EPIC): study populations and data collection. Public Health Nutr..

[52] González CA (2004). El estudio prospectivo europeo sobre cáncer y nutrición (EPIC)(#). Rev. Española de. Salud Pública.

[53] Riboli E, Kaaks R (1997). The EPIC project: rationale and study design. European Prospective Investigation into Cancer and Nutrition. Int. J. Epidemiol..

[54] Naz S (2015). Unveiling differences between patients with acute coronary syndrome with and without ST elevation through fingerprinting with CE‐MS and HILIC‐MS targeted analysis. Electrophoresis.

[55] Garcia, A. & Barbas, C. Gas chromatography-mass spectrometry (GC-MS)-based metabolomics. *Methods Mol. Bio. Springer* **708**, 191–204 (2011). 10.1007/978-1-61737-985-7_11 21207291

[56] Ciborowski M (2010). Metabolomic approach with LC–MS reveals significant effect of pressure on diver’s plasma. J. Proteome Res..

[57] Dudzik, D., Barbas-Bernardos, C., García, A. & Barbas, C. Quality assurance procedures for mass spectrometry untargeted metabolomics. a review. *J. Pharm. Biomed. Anal.* **147**, 149–173 (2017). 10.1016/j.jpba.2017.07.044 28823764

[58] Armitage EG, Godzien J, Alonso‐Herranz V, López‐Gonzálvez Á, Barbas C (2015). Missing value imputation strategies for metabolomics data. Electrophoresis.

[59] Kuligowski J, Sánchez-Illana Á, Sanjuán-Herráez D, Vento M, Quintás G (2015). Intra-batch effect correction in liquid chromatography-mass spectrometry using quality control samples and support vector regression (QC-SVRC). Analyst.

[60] de la Fuente AG (2018). Knowledge-based metabolite annotation tool: CEU mass mediator. J. Pharm. Biomed. Anal..

[61] Gil-De-La-Fuente A (2019). CEU mass mediator 3.0: a metabolite annotation tool. J. Proteome Res..

[62] Barberini L (2016). Multivariate data validation for investigating primary HCMV infection in pregnancy. Data Brief..

