# Supplementary material for: Prognostic biomarkers of Parkinson’s disease in the Spanish EPIC cohort: a multiplatform metabolomics approach
Source: NPJ Parkinsons Dis. 2021 Aug 16;7:73. doi: 10.1038/s41531-021-00216-4 (PMC8368017; doi:10.1038/s41531-021-00216-4)
Supplement: Supplementary file 1 — Supplementary Information [file 41531_2021_216_MOESM1_ESM.pdf]

Prognostic biomarkers of Parkinson´s Disease in the Spanish EPIC cohort: a multiplatform metabolomics approach

Carolina Gonzalez-Riano [1], Jorge Sáiz [1], Coral Barbas [1*], Alberto Bergareche [2,3,4*], José Mª Huerta [5,6], Eva Ardanaz [6,7], Marcela Konjevod [8], Elisabet Mondragon [2], ME Erro [9], M. Dolores Chirlaque [5,6], Eunate Abilleira [10], Fernando Goñi-Irigoyen [6,10], Pilar Amiano [6,10]

[1] Centro de Metabolómica y Bioanálisis (CEMBIO), Facultad de Farmacia, Universidad San Pablo-CEU, CEU Universities, Urbanización Montepríncipe, Boadilla del Monte, 28660 Madrid, Spain

[2] Neurodegenerative Disorders Area, Biodonostia Health Research Institute, San Sebastián, Spain

[3] Disorders Unit, Department of Neurology, University Hospital Donostia, San Sebastián, Spain

[4] Biomedical Research Networking Centre Consortium for the area of Neurodegenerative Diseases (CIBERNED), Madrid, Spain

[5] Instituto Murciano de Investigación Biosanitaria (IMIB)

[6] CIBER de Epidemiología y Salud Pública (CIBERESP), Madrid, Spain

[7] Instituto de Salud Pública de Navarra, Pamplona, Spain

[8] Rudjer Boskovic Institute, Division of Molecular Medicine, Bijenicka cesta 54, 10000 Zagreb, Croatia

[9] Department of Neurology. Complejo Hospitalario de Navarra, IdiSNA (Navarra Institute for Health Research), Pamplona, Spain

[10] Public Health Laboratory in Gipuzkoa, Biodonostia Health Research Institute, San Sebastián, Spain

* Authors to whom correspondence should be addressed:

e-mail address: cbarbas@ceu.es
e-mail address: JESUSALBERTO.BERGARECHEYARZA@osakidetza.eus

SUPPLEMENTARY NOTE 1

Chemicals

Organic solvents (MS grade), analytical grade formic acid 99%, standard mix for GC-MS containing grain fatty acid methyl ester (FAME) mixture (C8:0–C22:1n9), methanol, ethanol, and tricosane were from Sigma-Aldrich. Analytical grade heptane was purchased from Fluka Analytical (Sigma-AldrichChemie GmbH, Steinheim, Germany). Sialylation-grade pyridine was from VWR International BHD Prolabo (Madrid, Spain). Reference mass solutions for LC-MS and CE-MS were from Agilent Technologies. Ultrapure water (Milli-Qplus185 system Millipore, Billerica, MA, USA) was used in preparation of all buffers and standard solutions. The palmitic acid, stearic acid, palmitoleic acid, oleic acid, linoleic acid, and arachidonic acid standards were purchased from Sigma-Aldrich (Steinheim, Germany).

Metabolites extraction for Liquid chromatography coupled with mass spectrometry (LC-QTOF-MS and LC-QqQ-MS) analyses

The 78 randomly selected plasma samples (pre-PD group, *n* = 39; control group, *n* = 39; gender balanced) were thawed on ice for approximately 1 hour. The samples were vortex-mixed for 2 minutes and 100 µL of plasma were transferred to an Eppendorf tube. Subsequently, 300 µL of a previously prepared cold mixture (-20°C) of methanol:ethanol (1:1, v/v) were added in the Eppendorf tube for deproteinization. After stirring the samples for 1 minute, they were incubated on ice for 5 minutes and vortex-mixed for another minute. Samples were centrifuged for 20 minutes at 13,000 rpm at 4 °C. After centrifugation, 100 µL of the supernatant was transferred to a chromatography vial with insert and was directly injected into the system.

Metabolites extraction for Gas chromatography coupled with mass spectrometry analysis

Once the samples were thawed on ice for 1 hour, they were vortex-mixed for 2 minutes and 40 µL of plasma were transferred to an Eppendorf tube. A volume of 120 µL of cold acetonitrile (-20 °C) was used for deproteinization. Sample preparation continued with vortex mixing for 2 minutes, incubation on ice and centrifugation for 10 minutes at 15,400 rpm at 4°C. Then, 100 µL of the supernatant were transferred to a GC-MS vial to be evaporated to dryness in a vacuum concentrator. Once the vials were completely dry, the derivatization process was continued to obtain volatile derivatives for analysis. First, 10 µL of *O*-methoxyamine in pyridine (15mg/mL) were added to each of the vials. The samples were then vortex-mixed vigorously for 5 minutes, and then 3 sonication cycles (2 minutes) and 3 vortex cycles (2 minutes) were performed. The samples were covered with aluminum foil and incubated at room temperature, in the dark to complete the methoximation process. After 16 hours of incubation, 10 µL of N,O

bis(trimethylsilyl)trifluoroacetamide (BSTFA) with 1% trimethylchlorosilane (TMCS) were added to all the samples, followed by 5 minutes of vortex-mixing. Samples were placed in the oven at 70 °C for 1 hour to complete the silylation reaction, which was followed by 30 minutes of cooling down. Finally, 100 µL of heptane with 20 ppm of tricosane (internal standard) were added to the samples and mixed for 1 minute in the vortex mixer.

Metabolites extraction for Capillary electrophoresis coupled with mass spectrometry analysis

After thawing the samples on ice for 1 hour, they were vortex-mixed for 1 minute and 100 µL of plasma were transferred to an Eppendorf tube. Subsequently, 100 µL of 0.2 M formic acid containing 5% acetonitrile and 0.4 mM methionine sulfone as an internal standard were added. The samples were vortex-mixed for 1 minute and filtered with a Millipore filter to remove proteins. Finally, the samples were centrifuged for 70 minutes, at 2000 rpm, at 4 °C. After centrifugation, 90 µL of the supernatant were transferred to a CE-MS vial for analysis.

Analytical settings for the UHPLC-QTOF-MS analysis

The analysis of the samples was accomplished using an UHPLC system (1200 Infinity system, Agilent Technologies, Waldbronn, Germany), coupled to a 6520 QTOF MS (Agilent Technologies) with an ESI ion source. The sample injection volume was set up to 10 µL. The separation was achieved using a Discovery® HS C18 15cm x 2.1 mm, 3 µm (Supelco analytical) reverse phase column at thermostated 40 °C. The gradient used for the analysis consisted of a mobile phase A (0.1% formic acid in Milli-Q water) and a mobile phase B (0.1% formic acid in acetonitrile) pumped at 0.6 mL/min. The chromatography gradient began with 25% of phase B, increasing to 95% B in minute 35. The gradient then decreased to 25% of B in minute 36 and was maintained for 9 minutes until minute 45. Data were collected in positive and negative ESI modes in separate analyses, operated in full scan mode with a mass range of 50 to 1000 m/z for both modes. The capillary voltage was set to 3500 V for positive and 4000 V for negative ionization mode, the drying gas flow rate was 10.5 L/min at 330 ° C, gas nebulizer at 52 psi, and the fragment voltage 175 V. Two reference masses were used per ionization mode in order to provide a constant mass correction: m/z 121.0509 and m/z 922.0098 for the positive ionization mode, and m/z 119.0363 and m/z 966.0007 for the negative mode [4, 5].

Analytical settings for the GC-MS analysis

An Agilent GC system (7890A) coupled to a- 5975C mass spectrometer (Agilent Technologies) was used to perform metabolite fingerprinting of plasma samples. Briefly, 2 µL of derivatized

samples were automatically injected in split mode (ratio 1:10) through a split liner of ultra-inert deactivated glass wool from Agilent. The separation of the compounds was achieved using a pre-column (10 m J&W integrated with Agilent 122-5532G) combined with a GC DB5-MS column (length, 30 m; internal diameter, 0.25 mm; and 0.25 µm film of 95% of dimethyl/5% diphenylpolysiloxane). The flow rate of the carrier gas (helium) was constant at 1 mL/min through the column. The retention time (RT) was locked according to the peak of the internal standard (C18 methyl stearate) at 19.66 minutes. The temperature of the column was initially set at 60 °C for 1 minute, then raised to 10 °C/min to 325 °C, which was maintained for 10 minutes before cooling. The injector and transfer line temperatures were set at 250 °C and 280 °C, respectively. The operating parameters of electronic impact ionization were established as follows: filament source temperature at 230 ° C and electronic ionization energy at 70 eV. Mass spectra were collected in a mass range of 50 to 600 m/z at a scan rate of 2 spectra per second. Data was acquired using Agilent MSD ChemStation software (Agilent Technologies). To determine the retention rate, a mixture of n-alkanes (C8-C28) dissolved in n-hexane was analyzed before the samples.

Analytical settings for the CE-MS analysis

The analysis was performed using a 7100 capillary electrophoresis (Agilent Technologies) coupled to a TOF MS 6224 mass spectrometer (Agilent Technologies), equipped with an ESI ion source. For the separation of metabolites, an Agilent Technologies fused silica capillary (total length, 96 cm; internal diameter, 50 µm) was used, working in normal polarity. Before each analysis, the capillary was washed for 5 min (950 mbar) with background electrolyte (BGE) (0.8 M formic acid solution in 10% methanol (v/v)). The sample injection was performed during 50 s at 50 mbar and, in order to improve the reproducibility of the analysis, the BGE was injected for 20 s at 100 mbar after the injection of each sample. The separation was performed with an internal pressure of 25 mbar at a voltage of +30 KV and at a constant temperature of 20 ° C. The total analysis time was 30 minutes. Mass spectrometry was operated in positive polarity, with a mass range 74–1000 m/z at a scanning speed of 1.00 spectrum /s. Other parameters fpr the MS were: fragmentor at 100 V, skimmer at 65 V, OCT RF Vpp at 750 V, drying gas temperature at 250 °C, flow at 10 L/min, nebulizer at 4 psig and capillary voltage at 4000 V. The sheath liquid used consisted of methanol:water (1:1, v/v), formic acid (1 mM) and two reference masses (5 µL of purine: 121.0509 and 15 µL of HP-0921: 922.0098) at a flow rate of 0.4 mL/min (1:100 of split ratio).

Analytical settings for the HPLC-ESI-QqQ-MS analysis

The analysis of the samples was conducted using an HPLC system (1200 Infinity system, Agilent Technologies, Waldbronn, Germany), coupled to a 6470A QqQ MS (Agilent Technologies) with an ESI ion source. The sample injection volume was set up to 10 µL. The separation was achieved using a Zorbax SB-C18 (2.1x50 mm, 1.8 µm) (Agilent Technologies) reverse phase column at thermostated 55 °C. The gradient used for the analysis consisted of a mobile phase A (15 mM ammonium acetate) and a mobile phase B (15 mM ammonium acetate in methanol) pumped at 0.6 mL/min. The chromatography gradient began with 0 to 15 min from 20% B to 100% B, while post-time was set at 5 min. Data was collected in MRM in negative ESI mode. The capillary voltage was 3500 V. The nebulizer was set to 45 psi, the gas temperature 300 ºC and the flow rate was 5 L/min. The sheath gas temperature and flow rate were 250 ºC and 11 L/min respectively. Quantification was performed by the stable isotope dilution method. The data acquired was reprocessed with MassHunter Quantitative Analysis version B.09.00.

REFERENCES

1. González, C.A.*, et al.* El estudio prospectivo europeo sobre cáncer y nutrición (EPIC)(#). *Revista española de salud pública* **78**, 167-176 (2004).
2. Riboli, E. & Kaaks, R. The EPIC project: rationale and study design. European Prospective Investigation into Cancer and Nutrition. *International journal of epidemiology* **26**, S6 (1997).
3. Riboli, E.*, et al.* European Prospective Investigation into Cancer and Nutrition (EPIC): study populations and data collection. *Public health nutrition* **5**, 1113-1124 (2002).
4. Godzien, J.*, et al.* Rapid and reliable identification of phospholipids for untargeted Metabolomics with LC–ESI–QTOF–MS/MS. *Journal of Proteome Research* **14**, 3204-3216 (2015).
5. Whiley, L., Godzien, J., Ruperez, F.J., Legido-Quigley, C. & Barbas, C. In-Vial Dual Extraction for Direct LC-MS Analysis of Plasma for Comprehensive and Highly Reproducible Metabolic Fingerprinting. *Analytical Chemistry* **84**, 5992-5999 (2012).
